# A Long Road to Personalized Medicine in Heart Failure and Cardiomyopathies

**DOI:** 10.3390/jpm13010141

**Published:** 2023-01-11

**Authors:** Yuji Nagatomo

**Affiliations:** Department of Cardiology, National Defense Medical College, 3-2 Namiki, Tokorozawa 359-8513, Japan; con401@ndmc.ac.jp; Tel.: +81-4-2995-1597; Fax: +81-4-3996-5200

Over the past few decades, a drastic increase in the prevalence of heart failure (HF) has been observed worldwide, and is now often referred to as the “Heart failure pandemic”. Despite recent advances in the diagnosis and treatment of HF, its prognosis remains poor. In particular, cardiomyopathy exhibits the highest percentage of etiologies of advanced HF requiring cardiac transplantation. In this Special Issue, many researchers provided their latest findings regarding the diagnosis, risk stratification and treatment of HF and cardiomyopathies, which all may potentially lead to future personalized disease approaches.

Secondary cardiomyopathy is still a major challenge in terms of its diagnosis and treatment. Amyloid cardiomyopathy used to be considered to be a relatively rare disease, but now, is increasingly being recognized as a cause of left ventricular (LV) hypertrophy. Notably, transthyretin amyloid cardiomyopathy (ATTR-CM) comprises a not negligible proportion of HF in the elderly and presents as rapidly progressive HF, leading to a poor prognosis. Recently, novel drugs for ATTR-CM have emerged and can be prescribed to patients in clinical practice; hence, a timely diagnosis is crucial. Nakano et al. demonstrated the correlation of the extent of TTR deposition with LV diastolic dysfunction as a marker of reactive oxygen species [1]. This paper further adds to the evidence regarding the involvement of ATTR deposition in cardiac impairment. In the future, these findings have the potential to lead to more precise risk stratification for ATTR-CM patients. Furthermore, these findings may be informative for disease monitoring and valuable to guide treatment if there are multiple treatment options based on the severity of the disease.

Arrhythmogenic right ventricular cardiomyopathy (ARVC) is a rare condition of cardiomyopathy arising from a genetic disorder. The diagnosis of ARVC is sometimes challenging and difficult to differentiate from dilated cardiomyopathy. Magnetic resonance imaging (MRI) is a tool to help with the diagnosis of cardiomyopathies including ARVC. Manole et al. summarized the problem and limitations of the currently available criteria for ARVC and the additive value of cardiac MRI for a better diagnosis and risk stratification [2].

Over the last two decades, the progress in regard to pharmacological and nonpharmacological therapies has caused the dramatic improvement in survival and cardiovascular outcomes for HF with a reduced ejection fraction (HFrEF). Particularly, guideline-directed medical therapy (GDMT) is a cornerstone of HFrEF treatment. On the other hand, the insufficient use and titration of GDMT has been indicated in clinical practice. Among GDMTs, β-blocker therapy is one of the most potent drugs used to improve the symptoms and clinical outcomes for HFrEF. The mechanisms mediating its beneficial effects include, but are not limited to, the amelioration of sympathetic nerve overactivation, an anti-ischemic effect, an anti-arrhythmic effect, promoted reverse remodeling and the prevention of sudden cardiac death. Since the mortality benefit caused by β-blocker therapy was shown to be correlated with the reduction in heart rate, it can be determined that heart rate is an important hallmark of β-blockers’ effect. In this context, questions have been raised. Do we need a maximum dose of β-blockers for all patients? If this is the case, what should we do if the heart rate goes too low? These questions converge into the next question: what is the optimal heart rate? However, the target heart rate (THR) we should aim for has not been thoroughly investigated. In order to answer this question, Yumita et al. focused on the overlap between E and A waves in mitral inflow and they hypothesized that a heart rate which yields no E-A overlap might be hemodynamically ideal and could be a reasonable target [3]. This THR nicely discriminated between long-term clinical events more precisely than a uniform heart-rate cutoff (i.e., 70 bpm). Although this “THR theory” is not fully established, THR, which is determined on a per-patient basis, will pave the way for the use of personalized therapeutic strategies for HFrEF, including cardiomyopathies.

As a nonpharmacological therapy, cardiac resynchronization therapy (CRT) is a therapeutic strategy efficacious against advanced HFrEF with dyssynchronous LV motion. There has been a long debate on its indication and responder prediction. The personalized CRT setting after implantation has also been under debate, but there has been no consensus. Leitz et al. demonstrated the extended findings of their prospective observational study, where they followed their study patients for as long as approximately 10 years [4]. In this paper, besides the New York Heart Association Functional Classification, etiology (ischemic), and CRT response, a wide QRS during biventricular pacing was proven to be associated with a higher mortality. Extending these findings, the potential benefit of utilizing the personalized CRT setting to aim for a narrower QRS during biventricular pacing may be proposed for better survival. The ongoing evolution of CRT implantation strategies and CRT devices can further facilitate the ideal CRT setting on a per-patient basis. However, these findings need to be confirmed by future studies.

Within human bodies, there are trillions of bacterial organisms belonging to >2000 species, the vast majority being in the gut. There is increasing evidence that the nature of the microbiome plays an important role in human health and disease. On the other hand, accumulating evidence suggests that the interaction between multiple organs plays a crucial role in the pathology of various diseases, including HF. In this context, the interaction between heart and gut, or the “gut-heart axis”, has emerged as a novel concept to provide new insights into the intricate mechanisms of HF. Since gut microbiota is highly variable from person to person, the microbiome approach to HF or cardiomyopathy potentially offers a new possibility for personalized medicine. Short-chain fatty acids (SCFAs) are gut microbiota-derived substances which might mediate the microbiota-driven pathology, leading to various organ dysfunctions. Yukino-Iwashita et al. summarized the current knowledge and perspectives on the possible role of SCFAs in the pathology of HF [5]. SCFAs are shown to have beneficial effects on the heart via their actions on blood pressure control, the immune system, adipose tissue, kidneys and the gut. Conversely, SCFA abundance can be modulated by the condition of the gut and/or heart. Collectively, SCFAs play a central role of mediating the “gut-heart axis” in HF. Although most of the proposed mechanisms of SCFAs that contribute to the pathology of HF shown in this paper are still under debate, and hypotheses are still being generated, microbiome research and SCFAs have the potential to open up a new research direction for a personalized approach to HF.

As a Guest Editor, I would like to thank all authors and reviewers for their contribution to this Special Issue and for the quality of submitted papers. I hope the presented novel findings will facilitate further developments and the refinement of personalized medicine in this area.
